# Complete Genome Sequence of Highly Virulent *Haemophilus parasuis* Serotype 11 Strain SC1401

**DOI:** 10.1128/genomeA.00628-16

**Published:** 2016-07-21

**Authors:** Ke Dai, Jin Jin, Yiping Wen, Xintian Wen, Lvqin He, Sanjie Cao, Xiaobo Huang, Rui Wu, Qin Zhao

**Affiliations:** Research Center of Swine Disease, College of Veterinary Medicine, Sichuan Agricultural University, Chengdu, China

## Abstract

*Haemophilus parasuis*, a normal Gram-negative bacterium, may cause Glässer’s disease and pneumonia in pigs. This study aims to identify the genes related to natural competence of the serotype 11 strain SC1401, which frequently shows competence and high pathogenicity. SC1401 shows many differences from strains without natural competence within the molecular basis. We performed complete genome sequencing together with restriction modification system analysis to lay the foundation for later study.

## GENOME ANNOUNCEMENT

*Haemophilus parasuis*, a typically NAD-dependent commensal bacterium found in the upper respiratory tract of swine (1), can invade the organism and cause a system syndrome called Glässer’s disease characterized by polyarthritis, fibrinous polyserositis, and meningitis, resulting in serious economic losses (2). 15 serotypes have been identified, in which serovars 1, 5, 10, 12, 13, and 14 show severe virulence and may cause death within 4 days (3, 4). To date, 22 genomes of *H. parasuis* have been promulgated inclusive of the strain SC1401, which is the first serotype 11 with its complete genome released.

Five strains with experimentally high frequencies of natural competence among 127 wild strains of *H. parasuis* isolated from China were found. Of these five strains, SC1401 was shown to have the relatively highest virulence strain. Serotype identity of SC1401 was confirmed by the Research Center of Swine Disease, College of Veterinary Medicine, Sichuan Agricultural University according to the PCR assay for molecular serotyping on the basis of variation within the capsule loci (5). Screening of bacteria is vital for studies of natural competence.

Whole-genomic DNA was extracted from SC1401 using the TIANamp bacteria DNA kit (Qiangen). The genome of SC1401 was sequenced on Pacbio (Beijing Novogene Bioinformatics Technology Co., Ltd.) by single molecule real-time (SMRT) technology (6) to obtain 38,882 reads and 180× sequencing-depth data. The filtered reads were assembled to generate one contig without gaps. Fifty-nine tRNA genes were predicted with tRNAscan-SE (7), 19 (5s-7,16s-6,23s-6) rRNA genes were predicted with rRNAmmer (8), and 2 small RNAs (sRNAs) were predicted by BLAST against the Rfam (9) database. Repetitive sequences were predicted using RepeatMasker (10) (http://www.repeatmasker.org/) to find 111 interspersed repetitive sequences. Tandem repeats were analyzed using Tandem Repeat Finder (11) (http://www.pathogenomics.sfu.ca/islandviewer/resources.php) to find 272 tandem repeat sequences. PHAST (12, 13) was used for prophage prediction (http://phast.wishartlab.com/) and CRISPRFinder (13) for clustered regularly interspaced short palindromic repeat (CRISPR) identification.

Gene prediction was performed on the SC1401 genome assembly by GeneMarks (14) (http://topaz.gatech.edu/). To predict protein-coding genes, a whole-genome BLAST search was performed against 6 databases including KEGG, COG, nr, Swiss-Prot, GO, and TrEMBL. Pathogenicity and drug resistance was analyzed searching 4 databases: PHI (pathogen host interactions), VFDB (virulence factors of pathogenic bacteria), ARDB (antibiotic resistance genes), and CAZy (carbohydrate-active enzymes). Secretory proteins were detected on the genome assembly by SignalP (15). Type I-VII secretion system related proteins were extracted from all the annotation results. Type III secretion system effector proteins were detected by EffectiveT3 (16). Secondary metabolite gene clusters were predicted by antiSMASH (17).

The whole-genomic analysis showed a genome size of 2,277,540 bp, with a mean G+C content of 40.03%. The total gene number is 2,234, accounting for 87.65% of the whole genome. Epigenetic modification prediction shows 4,094 loci of type m4C and 23,735 loci of type m6C.

### Nucleotide sequence accession numbers.

This genome sequence has been deposited at DDBJ/EMBL/GenBank under the accession number CP015099. The version described in this paper is the first version CP015099.1.
